# An Observational Cohort Comparison of Facilitators of Retention in Care and Adherence to Anti-Eetroviral Therapy at an HIV Treatment Center in Kenya

**DOI:** 10.1371/journal.pone.0032727

**Published:** 2012-03-09

**Authors:** Loice Achieng, Helen Musangi, Sharon Ong'uti, Edwin Ombegoh, LeeAnn Bryant, Jonathan Mwiindi, Nathaniel Smith, Philip Keiser

**Affiliations:** 1 A.I.C. Kijabe Hospital, Kijabe, Kenya; 2 University of Nairobi, Nairobi, Kenya; 3 Johns Hopkins University, Baltimore, Maryland, United States of America; 4 University of Texas Medical Branch, Galveston, Texas, United States of America; 5 Arkansas Department of Health, Little Rock, Arkansas, United States of America; Boston University, United States of America

## Abstract

**Background:**

Most HIV treatment programs in resource-limited settings utilize multiple facilitators of adherence and retention in care but there is little data on the efficacy of these methods. We performed an observational cohort analysis of a treatment program in Kenya to assess which program components promote adherence and retention in HIV care in East Africa.

**Methods:**

Patients initiating ART at A.I.C. Kijabe Hospital were prospectively enrolled in an observational study. Kijabe has an intensive program to promote adherence and retention in care during the first 6 months of ART that incorporates the following facilitators: home visits by community health workers, community based support groups, pharmacy counseling, and unannounced pill counts by clinicians. The primary endpoint was time to treatment failure, defined as a detectable HIV-1 viral load; discontinuation of ART; death; or loss to follow-up. Time to treatment failure for each facilitator was calculated using Kaplan-Meier analysis. The relative effects of the facilitators were determined by the Cox Proportional Hazards Model.

**Results:**

301 patients were enrolled. Time to treatment failure was longer in patients participating in support groups (448 days vs. 337 days, P<0.001), pharmacy counseling (480 days vs. 386 days, P = 0.002), pill counts (482 days vs. 189 days, P<0.001) and home visits (485 days vs. 426 days, P = 0.024). Better adherence was seen with support groups (89% vs. 82%, P = 0.05) and pill counts (89% vs. 75%, P = 0.02). Multivariate analysis using the Cox Model found significant reductions in risk of treatment failure associated with pill counts (HR = 0.19, P<0.001) and support groups (HR = 0.43, P = 0.003).

**Conclusion:**

Unannounced pill counts by the clinician and community based support groups were associated with better long term treatment success and with better adherence.

## Introduction

The roll out of anti-retroviral therapy in resource-limited setting has resulted in millions of individuals now receiving life saving therapy [1]. The U.S. Presidents Emergency Plan for AIDS Relief was initially envisioned as an emergency response to a humanitarian crisis. As anti-retroviral medications are reaching increasing numbers of HIV infected persons, the focus of this program has shifted to sustaining these efforts [2]. Sustainability of systems of HIV care has many facets but retention in care and adherence to anti-retroviral medication are critical to the maintenance of these endeavors.

There is a need to identify effective methods to retain patients in care and promote adherence to HIV regimens [3]–[6]. A recent meta-analysis of published reports found that only 60% of patients started on anti-retroviral drugs remained on therapy after 2 years [4]. Identification of approaches that optimize adherence to therapy remains a key challenge [7]. Data from developed countries have shown that missing 10% to 15% of doses of anti-retroviral drugs is linked to incomplete suppression of viral replication, declining CD4 cell counts, progression to AIDS, and the emergence of antiretroviral drug resistance [8], [9].

In sub-Saharan Africa, a number of evidence based interventions to retain patients in care and to foster adherence to HIV medications have been reported [10]–[20]. These interventions were designed to address stigma, isolation, lack of community support and poor health literacy, all of which have all been shown to contribute to poor adherence retention into care. Community based programs are widely used to support patients in taking anti-retroviral drugs, encourage attendance at clinics and to find treatment defaulters [11], [12], [14], [18], [20]. Intensive patient education by community health workers, pharmacists and clinical care providers is used both before and during therapy [15], [17], [20]. “Real Time” monitoring of pill taking by clinicians and pharmacists can also identify individuals with adherence difficulties that may require additional counseling [10], [14], [21]. [1] Most program evaluations are uni-dimensional and do not examine the overlapping effect of the multiple approaches to improve retention into care and anti-retroviral adherence. Similarly, many studies focus on a single endpoint and do not evaluate other important outcomes such as retention in the program, virologic success and survival. Etienne et al studied a tiered approach to retention in care and adherence in 27 countries and found that those centers that utilized community programs, intensive adherence education and active monitoring of pill taking had the best results when compared to centers that only offered basic patient education [22].

We performed a prospective, observational cohort analysis of a single medical center that utilizes tiered approach retention in care and adherence promotion [22]. As a precondition to participation into the program, patients agree to participate all of the treatment and adherence activities. In reality, many patients only participate in some, allowing for an evaluation of the relative, individual contribution of each of the interventions on the long-term patient outcomes through a multivariate analysis. Thus we can identify the most effective, easily scalable, practices that will support sustainability of the anti-retroviral effort in resource limited settings.

## Methods

### Study Setting

AIC Kijabe Hospital is a 265 bed hospital located 60 kilometers northwest of Nairobi, Kenya. It follows approximately 6000 patients on anti-retroviral therapy at six locations in Central Kenya. Kenya utilizes a catchment system of care for HIV patients and thus only those who live within a 40 km radius of the hospital are eligible for enrollment into the treatment program. The program utilizes a wide array of personnel to provide care that include consultant physicians, medical officers and clinical officers. Nurses assist in care in the clinics and coordinate community health workers. A community heath program using nurses and community health workers acts as a liaison between the clinics and the patients and manages community-based facilitators of adherence and retention into care. All medications are dispensed by the Kijabe pharmacy by a pharmacist. Medical records are abstracted and entered into an electronic database.

**Figure 1:**
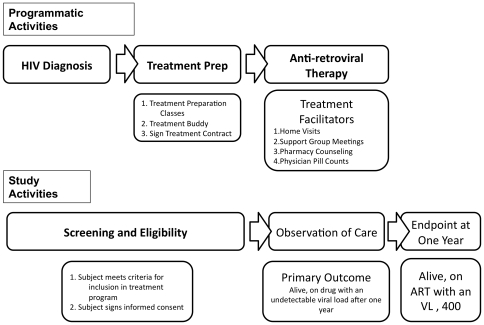
Schematic Diagram of HIV Program and Observational Study Design. The HIV program is divided into 3 distinct activities, HIV testing, Treatment Preparation, and Antiretroviral treatment that includes both community and clinic based facilitators. For entry into the study, subjects must be HIV positive and complete all treatment preparation activities. The study period begins with initiation of ART and evaluates the effects of treatment facilitators on treatment success.

The Kijabe HIV program utilizes the highest tiered approach to facilitate adherence and promote retention into care described by Etienne et. al. A schematic of the treatment program and of the study are shown in Figure 1. The nature of adherence support and training of staff are described in the prior publication. [22]. During the first six months of HIV therapy, patients must agree to participate in all adherence activities as a condition for receiving medication. Specific elements of the program include:

### Treatment Preparation Classes

Patients learn about HIV infection, treatment, the importance of adherence and of programmatic requirements. There are three required classes over 3 weeks, each lasting about one hour.

### Treatment Buddy

Patients are required to identify a treatment buddy who is typically someone who has already been in treatment to encourage and assist with medication adherence.

### Treatment Contract

Patients sign a contract agreeing to participate in both the community program and in the medical care provided by the program.

### Community Program

1. **Home Visits.** Within one month of initiating ART, community health workers visit patients in their homes and assess barriers to care, patient adherence to medication and overall health status. Patients are referred back to the clinic if acute medical problems are encountered. Repeat visits are scheduled each month as need.
2. **Support Groups Meetings.** Support group meetings are held monthly in each of the six local communities where there are clinic sites. Meetings are typically held in local churches. Community health workers organize the support group. Patients attend with their treatment buddies. There are open discussions of both problems and successes associated with HIV therapy.

### Medical Care

Patients must come to the clinic on a monthly basis for the first six months. They undergo a medical evaluation by their care provider.

1. **Unannounced Pill Counts.** Patient providers count pills to assess their adherence and to provide a context for adherence counseling.
2. **Pharmacy Counseling.** After the care provider visit, the patient meets with a pharmacist to discuss any difficulties in taking medication and to further address adherence issues discovered during the provider visits.

### Participants

Study participants were recruited from AIC Kijabe Hospital's HIV Program.

Eligibility requirements for the study were:

1. HIV positive by Kenyan National AIDS and STI Control Program Criteria.
2. Age greater than 18 years old.
3. Anti-retroviral naïve with the exception of women who had previously received medication as part of prevention of mother to child transmission.
4. Completion of anti-retroviral treatment preparation course.
5. Identification of a Treatment buddy.
6. Signed treatment contract.
7. Providing informed consent.

### Ethics

This study was approved by the A.I.C. (African Inland Church) Kijabe Hospital Ethics Board, and the Kenya Medical Research Institute National Ethics Committee and the University of Texas Medical Branch Institutional Review Board. All subjects provided written informed consent prior enrollment into the study. Consent forms were available in both English and Swahili. For subjects who could not read either language, the consent form was explained to them by native speakers in their mother tongues.

### Study Design/Intervention

This was a prospective, observational cohort study that did not involve an intervention.

### Objectives

The objective of this study was to compare the relative effect of participation in ongoing facilitators of retention into care and adherence that occur after treatment initiation on time to treatment failure. Specific facilitators evaluated were:

- Home visits
- Support Groups Meetings
- Pharmacy Counseling
- Un-announced Pill Counts

### Outcomes

The primary outcome was defined as time to treatment failure from date of initiation of anti-retroviral medication. Treatment failure was defined as any one of the following:

1. Virologic failure: defined as failure to achieve an HIV-1 RNA less than 200 copies/ml at 6 months or an HIV-1 RNA greater than 200 copies/ml at one year after achieving an undetectable viral load.
2. Lost to Follow Up: Defined as not appearing for any treatment program activities for 90 days and inability of the community health workers to find the subject.
3. Discontinuation of Therapy: Defined as not picking up prescriptions for ninety days.
4. Death: verified by clinical personnel or by community health workers performing home visits.

### Data Collection

Participants were followed for one year after initiation of ART. Data was primarily collected from the patient medical record. Demographic data was collected from participant charts at baseline. CD4 counts were performed at initiation of therapy following standard of care. Viral loads were measured on study participants at baseline, 6 months, and 12 months. Data was managed using Microsoft Access. Dispensation of anti-retroviral drugs was collected from months 1 to 12. Participation in each of the key activities of the study was collected at each month.

Kijabe utilizes patient treatment logs for both clinical and administrative purposes and these were used to confirm patient participation in study activities. All participants in support groups were required to sign an attendance sheet at the beginning of each meeting. Confirmation of home visits was confirmed by reviewing travel receipts for community health workers. Pharmacists confirm counseling visits in the dispensation record. Pill counts were solely recorded in the clinic record at the time the patient was seen by the clinician.

### Laboratory Methods

Blood samples were drawn from participants at baseline, 6 months, and 12 months. Serum was separated and stored in a −80 freezer for batched HIV-1 RNA testing. HIV-1 RNA testing was performed at AIC Kijabe Hospital using the ExaVir HIV-1 RT assay (Cavidi, Uppsala Sweden). This is an HIV-1 RT assay that correlates well with standard HIV-1 PCR and is widely used in resource-limited setting [23]. Samples were stored in a −20 freezer until they could be run in batches. HIV CD4 testing was performed using FACSCount (BD, UK).

### Sample Size

Preliminary data from a quality improvement project performed in 2008 found that 85% of HIV positive adults at Kijabe who had been on anti-retroviral therapy from 9–18 months had an undetectable HIV-1 viral load. The same study found a drop out rate of 10% for subjects who were initiated on anti-retroviral therapy. From this data, we assumed that 25% of subjects would be treatment failures in our study. Based on this, 280 subjects would provide an 80% power at a significance level of 0.05 when 5 independent variables are included; corresponding to an odds ratio of 2.**5**.

### Statistical Analysis

The primary endpoint was time to clinical failure. Failure was defined as being lost to follow-up, death, or having a detectable viral load at the end of study follow-up. The relationship between each patient activity and treatment success or failure was evaluated using Pearson's CHI squared test, Fishers exact test or student t test where appropriate. Adherence to medication was collected by review of pharmacy refill records for each participant using the method described by El-Khatib [24]. Time to failure was modeled using Kaplan-Meier plots.

The effects of participation in study activities on treatment failure were compared in two ways: first as a (Yes/No) and then a cut point between the differences in the medians between of treatment successes and failures. Those factors that were statistically significant (P<0.05) were further evaluated by comparing time to treatment failure. Significant differences between treatment failure and success were further evaluated using Cox Proportional Hazard regression models. The level of statistical significance utilized was p<0.05

## Results

Participant Flow is shown in Table 1. Subjects were enrolled between November 1, 2009 and April 20, 2010. There were 351 subjects screened. Of these 50 (14%) were ineligible because of age, prior ART or a subsequent negative HIV test. This latter group is of interest because all came to the study with reports of positive HIV test. Repeat HIV testing were negative in all of these including a negative HIV viral load. There were 301 subjects enrolled. Of these, 10 transferred out of care and their data was censored. Of the remaining 291 subjects, 85 (29%) were treatment failures and 206 were treatment successes. Only 40% of subjects participated in all of the required adherence activities.

**Table 1:** Participant Flow and Outcomes.

	N (%)
**Patients Screened**	351 (100)
Ineligible	50
Negative HIV Test	13
Prior ART	3
Age<13	34
**Enrolled**	301
Outcomes	
Virologic Failure	48 (15.9)
Dead	15 (5)
Lost to Follow Up	22 (7.3)
Transferred Out	10 (3.3)
Treatment Success	206 (68.4)

Table 2 compares the baseline characteristics between the treatment successes and treatment failures. There were no differences between the groups with regard to age at initiation of therapy, baseline CD4 cell counts, baseline viral load, employment status or distance to clinic. Treatment successes were more likely to be female (74% vs. 61%, P = 0.027, Pearsons Chi square test). Adherence as measured by pharmacy refill history was better in the treatment successes than the failures (90% vs. 82%, P = 0.043, Pearsons Chi square test). Successes were more likely to attend support group meetings (median 3 vs. 2, P = 0.001, Fisher's exact test), to have pharmacy counseling (median 2 vs. 1, P = 0.01, Fisher's exact test), to have a pill count performed by their clinician at a clinic visit (median 4 vs. 3, P = 0.001, Fisher's exact test) and to have attended all six of their clinic visits within the first 6 months of therapy (median 6 vs. 5, P = 0.001, Fisher's exact test). There was no difference in the median number of home visits between treatment successes and failures.

**Table 2:** Comparisons of Treatment Success and Failures.

Factors	Success(N = 206)	Failures(N = 85)	P Value
Age (mean)	37	37	0.97
CD4 Count (mean)	170 cells/mm3	170 cells/mm3	0.97
Viral Load (mean log_10_)	4.44	4.57	0.28
Female (%)	74%	61%	0.027
Unemployed (%)	43%	40%	0.58
Distance from Clinic (mean)	14.2 km	13.7 km	0.78
Adherence (mean)	90%	82%	0.043
**Interventions** (median)			
Home Visits	1	1	0.096
Support Groups	3	2	0.001
Pill Counts	4	3	0.001
Post Pharmacy Counseling	2	1	0.013
Clinic Visits	6	5	0.001
Total Number of Facilitators	5	4	0.083

**Figure 2:**
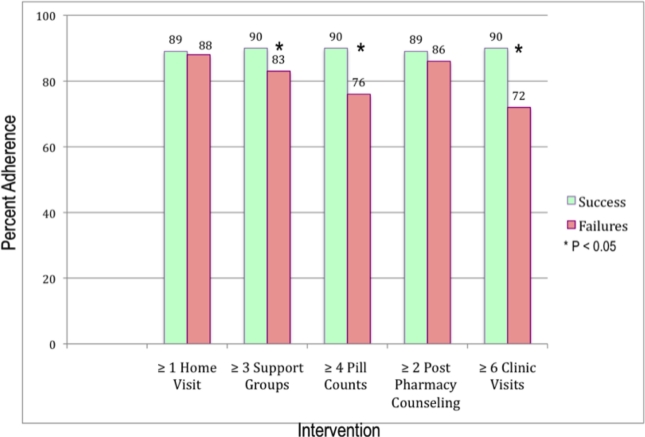
Rates of Adherence Categorized by Interventions. Rates of adherence in treatment successes and failures is shown by each intervention: 1) greater than one home visit; 2) participation in two or more support groups; 3) having four or more unannounced pill counts by primary care provider; 4) having two or more counseling sessions by a pharmacist; 5) completing six or more clinic visits.

Figure 2 compares the percent adherence between the treatment successes and failures by median number of patient activities. The cut points for each of the interventions were based on the medians shown in Table 2. Adherence was significantly higher in those who participated in 3 or more support group meetings (90% vs. 83%, P<0.05), who had pill counts performed by their clinician (90% vs. 76%, P = 0.001, and who attended all clinic visits (90% vs. 72% P = 0.001). Homes visits and pharmacy counseling were not associated with differences in adherence.

**Figure 3:**
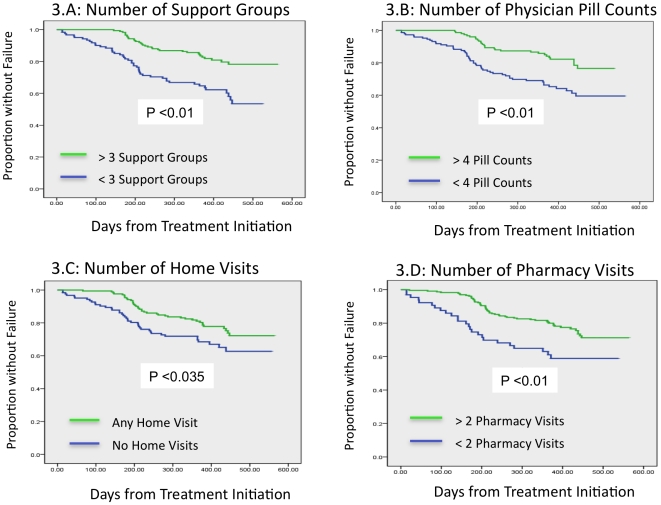
Time to Treatment Failure Categorized by Interventions. Kaplan Meier plots of time to treatment failure stratified by: A) participation in two or more support group meetings; B) having four or more unannounced pill counts by primary care provider; C) at least one home visit; D) having two or more counseling sessions by a pharmacist.

Time to treatment failure is shown in Figure 3. There was a significantly shortened time to treatment failure associated with having home visits (463 days vs. 394 days), having four or more pill counts by their provider (481 days vs. 355 days), participating in three or more support groups (480 days vs. 425 days), and participating in 2 or more pharmacy counseling sessions (461 days vs. 338 days). Each of these comparisons were statistically significant with P<0.05 by log-rank test.) However, when time to treatment failure was analyzed based on whether subjects had ever participated in any of the adherence activities (yes vs. no) only participation support groups, ever having a pill count performed or completing all six clinic visits were statistically significant. We analyzed the whether the total number of activities that the subjects participated in was predictive of outcome but could find no significant associations.

Multivariate analysis of the time to treatment failure using the Cox Proportional Hazards model is shown in Table 3. The baseline characteristics that were considered for inclusion into the model are listed in paragraph one and in Table 2. In addition, we also tested CD4 cell count, log of viral model as these have been previously shown to affect time to failure. In the multivariate analysis, CD4 and viral load were not significant and were not significant in comparison of baseline differences in outcomes and were ultimately not included in the model. Tests of co-linearity between clinic visits, pill counts, and pharmacy visits were not significant (p>0.05 for all comparisons) On multivariate analysis, there was a significant reduction in risk of failure with completion of all clinic visits (HR = 0.46, p = 0.003), participation in 3 or more support groups (HR = 0.54, p = 0.014) and having 4 or more pill counts performed by the subjects clinicians (HR = 0.57, p = 0.026).

**Table 3:** Multivariate Analysis of Risk of Failure.

Factor	Risk	P
≥6 clinic visits	0.46	0.003
≥3 Support Groups	0.54	0.014
≥4 Pill Counts	0.57	0.026
Female Gender	0.62	0.034

(Cox Proportional Hazards Model). P>0.05 for interactions.

## Discussion

There is an urgent need to identify effective facilitators of adherence and retention to care in resource limited settings. Observational studies can help identify potentially effective means of promoting successful treatment outcomes and can direct future investigations. Many studies are retrospective and/or use retention in care as the primary endpoint [3]–[6], [15], [25]–[27]. The high lost to follow up rates in such analysis had led to the call for better methods to assess vital status in resource limited settings [28]–[30]. In this study, we sought to determine the elements of a treatment program that were associated with successful HIV care. The setting for the study included both community and hospital based activities and utilized multiple, evidence based interventions to promote adherence to HIV care. Although subjects agreed to participate in all of these activities as a precondition to entry, only 40% of subjects were 100 percent compliant, thus allowing for an evaluation of the relative, individual contribution of each of the interventions on the long-term patient outcomes through a multivariate analysis. The prospective nature of this analysis meant that we were able to account for all but seven percent of the patients who enrolled in the study, a percentage that is significantly lower than reported in other studies. Our use of multiple viral load tests allowed for earlier recognition of failure of anti-retroviral medications. Had we solely relied on CD4 count declines, it may have taken several years before individuals had reached a clinical endpoint. Of the facilitators evaluated, we found participation in community based support groups, and unannounced pill counts performed by the patients care provider in front of the patient were associated with improved adherence and decreased risks of treatment failure. We also found that women were more likely to be successfully retained in care and treated, a finding that has been seen in other studies [16].

A significant finding of this study is that pill counts were associated with better treatment outcomes. Although pill counts have been widely studied as tool to measure adherence, there has been little evaluation of their use as facilitators of adherence. In our study, we examined only whether a pill count was performed but did not collect data on what the actual pill count was. We found that the number of times a pill count was performed as associated with both better adherence and better treatment outcomes, suggesting that intermittent pill counts may have a motivational effect on patients that result in better outcomes. In addition, pill counts are easy to perform, do not require special technology and are easily implemented across a wide spectrum of settings.

Our data has important implications for future research and for allocation of both clinical and research resources. Performance of pill counts by the clinician would seem to be a relatively easy and effective method for promoting adherence. Community based support groups require planning, personnel and travel, but also have a demonstrated clinical benefit. On the other hand, counseling by a pharmacist was not associated with clinical benefit nor did regular home visits seem to improve outcomes. The pharmacy counseling requires the time of a pharmacist and home visits require both community health workers time and travel. It may be that these interventions are not necessary for routine patient care and are best targeted for patients who have specific needs.

The results must be interpreted with caution. The study design is observational and thus there is potential for un-measured biases that could affect the outcome. An underlying assumption of the study was that each of the facilitators evaluated were independent predictors of treatment outcome. We were able to demonstrate associations between these interventions and did not find any interaction between significant facilitators on multivariate analysis. There was also no association between the total number of facilitators that subjects participated in and outcome, suggesting that the results were due to the individual factors evaluated and not to the total system of care. In addition, the study was performed within a single organization with multiple sites in rural Kenya and it is not clear how generalizable the results would be to other systems of care in other settings. We also did not perform a cost benefit analysis hence any discussion of cost effectiveness of the interventions would be speculative.

However, statistical associations do not demonstrate causality and further evaluations of our findings are warranted. The best way to evaluate the effectiveness of these interventions would be with a cluster-randomized trial using multiple sites. In such a design, one site would perform pill counts, another would perform have support groups in the community setting and a third would perform post clinic pharmacy counseling. The effect on clinical failure of each intervention would be determined. This would also allow for qualitative and quantitative assessments of the social, cultural and spiritual influencers on adherence and test interventions to address those influencers on the markers of failure and success.

A formal cost analysis could also be performed and the cost benefit of each intervention could be determined. Such a strategy would allow for the identification of the most cost effective interventions that could then be replicated in other settings.
